# Modified Fuzi Lizhong Decoction Alleviates Colonic Lesions and Modulates Gut Microbiota in Diabetic Gastroparesis

**DOI:** 10.2174/0118715303465130260619065650

**Published:** 2026-07-07

**Authors:** Xiaoxia An, Jinxiu Lan, Yongxiang Xie, Jiwu Li, Yan Wang, Lingmin Wu, Cui Liang, Shijuan Liu, Guangnuo Liu, Zhiqian Jiang, Zhengang Wang

**Affiliations:** 1 Department of Gastroenterology, The First Affiliated Hospital of Guangxi University of Chinese Medicine, Nanning City, Guangxi Zhuang Autonomous Region, 530023, China;; 2 Graduate School, Guangxi University of Chinese Medicine, Nanning City, Guangxi Zhuang Autonomous Region, 530023, China;; 3 Department of Nephrology, The First Affiliated Hospital of Guangxi University of Chinese Medicine, Nanning City, Guangxi Zhuang Autonomous Region, 530023, China;; 4 Department of Endocrinology, The First Affiliated Hospital of Guangxi University of Chinese Medicine, Nanning City, Guangxi Zhuang Autonomous Region, 530023, China

**Keywords:** Modified Fuzi Lizhong decoction, diabetic gastroparesis, intestinal mucosal barrier, gut microbiota, traditional Chinese medicine, rat model

## Abstract

**Introduction:**

Diabetic gastroparesis (DGP) is a debilitating complication of diabetes mellitus. This study investigated the therapeutic effects of Modified Fuzi Lizhong decoction (MFZLZD) on DGP.

**Methods:**

A DGP rat model was established through a high-fat diet combined with an irregular feeding schedule. Rats were treated with MFZLZD (5 or 20 g/kg) or mosapride (positive control; 1 mL/100 g) for 7 weeks. Body weight and gastric parameters (gastric emptying, peristalsis, and blood flow) were assessed. Histopathological alterations in gastric and colonic tissues were evaluated *via* HE staining, while goblet cell density was quantified by AB-PAS staining. The expression of tight junction proteins was analyzed using immunofluorescence. Finally, 16S rRNA sequencing of fecal samples was performed to characterize alterations in the gut microbiota.

**Results:**

MFZLZD significantly increased body weight and effectively restored gastric function in DGP rats. Moreover, MFZLZD treatment attenuated gastric and colonic mucosal injury, preserved goblet cell populations, and upregulated tight junction protein expression. Gut microbiota diversity was altered in DGP rats and was partially restored following MFZLZD treatment. Changes in 33 key microbial taxa were observed between groups.

**Discussion:**

MFZLZD improved gastric function and reinforced intestinal barrier integrity in DGP rats. The observed changes in gut microbiota composition suggest a potential association between alterations in the gut microbiota and the therapeutic effects of MFZLZD, although the underlying mechanisms remain unclear and require further investigation.

**Conclusion:**

MFZLZD represents a promising therapeutic strategy for DGP and is associated with improvements in intestinal barrier integrity, gastric function, and gut microbiota composition.

## 1 INTRODUCTION

Diabetes mellitus is a prevalent metabolic disorder primarily characterized by persistent hyperglycaemia [1]. Diabetic gastroparesis (DGP), a debilitating complication defined by impaired gastric motility and non-mechanical delayed gastric emptying, has an estimated prevalence of 9.3% among the diabetic population [2]. The pathogenesis of DGP is multifactorial, involving chronic hyperglycemia, autonomic neuropathy, ultrastructural disruption of the interstitial cells of Cajal, reduced nNOS levels, and gastrointestinal hormone imbalances [3]. While dietary modifications and prokinetic medications can alleviate DGP symptoms, their clinical utility is frequently constrained by adverse side effects [4]. Consequently, identifying novel, effective pharmacological interventions for DGP remains an urgent necessity.

The gut microbiota, an integral component of human physiology, comprises diverse populations of bacteria, archaea, eukaryotes, viruses, and parasites [5]. The gastrointestinal tract maintains bidirectional communication with the central nervous system *via* the microbiota-gut-brain axis; however, in DGP, microbial dysbiosis severely disrupts this regulatory axis [6]. Under diabetic conditions, gut microbiome perturbations lead to a diminished production of short- chain fatty acids (SCFAs), which subsequently compromises intestinal barrier integrity and increase mucosal permeability [7]. Additionally, the proliferation of proteolytic bacteria generates detrimental metabolites that exacerbate mucosal damage and metabolic dysfunction [7]. Clinical evidence indicates that probiotics can mitigate abdominal distension and accelerate gastric emptying in gastroparesis [8]. Zheng *et al*. reported that *Alpinia officinarum* Hance extract ameliorates DGP by reshaping gut microbiota [9]. Thus, targeting gut dysbiosis represents a promising therapeutic avenue for DGP.

Traditional Chinese medicine (TCM) decoctions have recently demonstrated favorable efficacy and safety profiles in the management of DGP [10]. Modified Fuzi Lizhong decoction (MFZLZD) is a classical TCM formula adapted from the original FZLZD documented in the ancient text “Sanyin Ji Yi Bingzheng Fang Lun”. MFZLZD comprises a synergistic blend of *Aconitum carmichaelii* Debeaux (Fuzi), *Codonopsis pilosula* Nannf. (Dangshen), *Atractylodes macrocephala* Koidz. (Baizhu), *Zingiber officinale* Roscoe (Ganjiang), *Poria cocos* Wolf (Fuling), *Magnolia officinalis* Rehd. et Wils. (HouPo), *Citrus aurantium* L., *fructus immaturus* (Zhishi), *Massa fermentata* (Shenqu), *Hordeum vulgare* L., fructus germinatus (Maiya), and *Salvia miltiorrhiza* Bunge (Danshen). Bai *et al*. found that MFZLZD exhibits superior efficacy compared to domperidone in stabilizing blood glucose and improving symptomatic outcomes in patients with DGP [11]. Furthermore, the Fuzi Lizhong Pill, which shares the same composition as FZLZD, has been shown to modulate gut microbiota in irritable bowel syndrome and ulcerative colitis [12, 13]. However, the specific regulatory effects of MFZLZD on gut microbiota associated with DGP remain to be elucidated.

In the present study, we utilized a DGP rat model to evaluate the therapeutic impact of MFZLZD on body weight, gastric histopathology, and colonic mucosal barrier. Moreover, 16S rRNA sequencing was employed to comprehensively analyze microbial shifts associated with MFZLZD treatment, aiming to uncover the underlying mechanisms of its therapeutic and protective effects.

## 2 METHODS

### 2.1 Preparation of MFZLZD

The dried herbs constituting MFZLZD were pulverized into a fine powder and passed through an 80-mesh sieve. A 50 g portion of the resulting powder was suspended in 500 mL of distilled water, followed by a 1 h maceration. The mixture underwent ultrasonic extraction at 40 kHz for 30 min and was subsequently heated at 80°C for 1 h. Following cooling and centrifugation, the supernatant was concentrated *via* thermal evaporation to a final concentration of 200 mg/mL for subsequent animal administration.

### 2.2 Animal Grouping

All animal experiments were conducted in accordance with the US National Research Council's “Guide for the Care and Use of Laboratory Animals”. Twenty-five male Sprague-Dawley rats (8 weeks old, 180-220 g) were obtained from Beijing HFK Biotechnology Co., Ltd. Rats were housed under controlled environmental conditions (18-25°C, 50-80% relative humidity, and 12-h light/dark cycle). After one week of adaptation, rats were randomly assigned to either the control group (n = 5), maintained on a standard chow diet, or the DGP group (n = 20), maintained on a high- fat diet. To induce DGP, streptozotocin (STZ; purity: 98.5%; Yuanye, KS397694) was dissolved in 0.1 mol/L citrate buffer to yield a 0.5% solution. Following four weeks of high-fat dietary intervention, the DGP group received a single intraperitoneal injection of STZ (40 mg/kg), while the control group received an equivalent volume of citrate buffer. Subsequently, DGP rats were randomly divided into four groups (n = 5): DGP, DGP + MFZLZD-L (5 g/kg), DGP + MFZLZD-H (20 g/kg), and DGP + mosapride (Mos, 1 mL/100 g; Aladdin, 112885-41-3) groups. The sample size for each group was determined based on previous studies [14]. Rats in the DGP, DGP + MFZLZD-L, DGP + MFZLZD-H, and DGP + Mos groups were subjected to an irregular feeding schedule (fasting on odd days and fed on even days) for 7 weeks. During this period, the treatment groups received daily oral gavage of the respective therapeutic agents. Body weights were recorded weekly, and rats were euthanized *via* CO_2_ inhalation at the end of the 11^th^ week. No adverse events were recorded during the study. Investigators were blinded to treatment groups during procedures and data analysis.

### 2.3 Gastric Emptying Analysis

Rats were administered 2 mL of 5% phenol red (Aladdin, 143-74-8) *via* oral gavage. After 30 min, rats were euthanized, and the stomach was isolated following ligation of the cardia and pylorus. Gastric contents were thoroughly recovered and mixed with 5 mL of 1 mol/L NaOH (Aladdin, 1310-73-2). Following a 1 h incubation, 5 mL of the supernatant was deproteinized using 20% trichloroacetic acid. After centrifugation at 3600 rpm for 10 min, the absorbance of the supernatant was measured at 560 nm using a spectrophotometer. The gastric emptying rate was calculated using the following formula: Gastric emptying rate = (1-measured absorbance/standard absorbance) × 100%.

### 2.4 Gastric Peristalsis Analysis

Rats were anesthetized *via* intravenous injection of propofol 30 min after the final administration. The stomach was surgically exposed, and the pyloric muscle was clamped to a muscle tension transducer under a baseline tension of 1 g. Once contractions stabilized, the gastric peristalsis curve was recorded for 10 min. The mean amplitude was calculated from three representative contraction cycles.

### 2.5 Gastric Blood Flow Analysis

Following anesthesia, the stomach was exposed, and a laser Doppler flowmeter probe was positioned near the lesser gastric curvature. After signal stabilization, gastric blood flow was recorded for 30 s, and the mean value from 10 s to 25 s was used for statistical analysis.

### 2.6 HE Staining

Gastric and colonic tissues were fixed in 4% formaldehyde (Beyotime, P0099) for 24 h, dehydrated, and embedded in paraffin (Sinopharm, 69019361). The paraffin blocks were sectioned at a thickness of 4-7 μm. Following deparaffinization and rehydration, sections were stained using an HE staining kit (Beyotime, C0105S) and visualized under a microscope (Leica, DM3000).

### 2.7 AB-PAS Staining

Colonic tissues were fixed in 10% neutral-buffered formalin for 24 h, dehydrated, and embedded in paraffin. Sections (4-7 μm thickness) were deparaffinized and incubated in periodic acid solution (Beyotime, C0142S-1) for 10 min. After rinsing, sections were stained with Schiff's reagent (Beyotime, C0142S-2) for 10 min, followed by counterstaining with hematoxylin (Beyotime, C0142S-3) for 3 min. Sections were dehydrated through a graded alcohol series and observed under a microscope.

### 2.8 Immunofluorescence

Colonic sections underwent heat-induced antigen retrieval at 92-96°C for 10-15 min. Sections were blocked with 5% BSA for 60 min and incubated overnight at 4°C with primary antibodies against ZO-1 (1:100; Abcam, ab307799) and Occludin (1:100; Abcam, ab216327). After washing, sections were incubated with a fluorophore-conjugated secondary antibody (1:200; Abcam, ab150077) and DAPI (Beyotime, C1005) for 60 min. Fluorescent signals were captured using a laser confocal microscope (Perkin Elmer, ULTRAVIEW VOX).

### 2.9 16S rRNA Microbiome Analysis

Genomic DNA was extracted from fecal samples and quantified using a NanoDrop ND-1000 spectrophotometer (Thermo Fisher Scientific, Wilmington, DE, USA). DNA integrity was verified by 1.0-1.5% agarose gel electrophoresis. The V3-V4 region of the 16S rRNA gene was amplified by PCR using the forward primer 5'-CCTACGGGNGGCWGCAG-3' and the reverse primer 5'-GACTACHVGGGTATCTAATCC-3'. Paired-end sequencing (250 bp) was performed on the Illumina NovaSeq 6000 platform. Raw sequences were filtered and denoised using DADA2 within the QIIME2 pipeline to generate an amplicon sequence variant (ASV) abundance table. The α diversity indices (ACE, Chao1, Shannon, and Simpson) and β diversity were subsequently assessed. LEfSe was employed to identify significant taxonomic differences between groups, using a threshold of LDA score > 2 and *P* < 0.05.

### 2.10 Statistical Analysis

Data are presented as mean ± standard deviation. Normality and homogeneity of variance were assessed using the Shapiro–Wilk test and Levene’s test, respectively. For normally distributed data with equal variances, one-way analysis of variance followed by Tukey's post hoc test was utilized for multiple group comparisons. For non-normally distributed data, the Kruskal-Wallis rank sum test followed by Dunn’s post hoc test was applied. All statistical analyses were conducted using GraphPad 7.0 software or R software (version 4.3.2). *P* < 0.05 was considered statistically significant. Each experiment was performed at least three times independently.

## 3 RESULTS

### 3.1 MFZLZD Alleviated the Symptoms of DGP Rats

Rats were maintained on a high-fat and irregular diet to establish the DGP model. During the initial four weeks of the dietary regimen, the DGP group exhibited a significant increase in body weight compared to the control group (Fig. **1A**). However, from the 4^th^ week onward, the body weight of the DGP rats progressively declined relative to controls (Fig. **1A**). Functional assessment of the stomach revealed that gastric emptying rate, peristalsis frequency, and gastric blood flow were significantly decreased in the DGP group (Fig. **1B**). Histological examination of the gastric tissues showed that in the control group, gastric glands and smooth muscle fibers were arranged in an orderly and compact fashion, with normal intercellular spaces (Fig. **1C**). In contrast, the DGP group displayed disorganized glandular structures, expanded intercellular spaces, reduced glandular cytoplasmic volume, vacuolar degeneration, and mucosal and submucosal congestion accompanied by vasodilation (Fig. **1C**). These phenotypic and pathological alterations confirmed the successful establishment of the DGP model. While no significant differences in body weight were observed among the DGP + MFZLZD-L, DGP + MFZLZD-H, and DGP + Mos groups during the first four weeks, the body weights of the treatment groups showed a gradual recovery from the 7^th^ week onward compared to the DGP model group (Fig. **1A**). Additionally, MFZLZD-H and Mos treatments partially improved gastric function (Fig. **1B**) and attenuated gastric tissue injury (Fig. **1C**). Given that MFZLZD improved DGP symptoms in a dose-dependent manner, subsequent experiments focused on elucidating the impact of MFZLZD-H on DGP.

### 3.2 MFZLZD Ameliorated Intestinal Mucosal Barrier Injury in DGP Rats

To determine whether the therapeutic efficacy of MFZLZD in DGP involves the modulation of the intestinal mucosal barrier, colonic tissue alterations were analyzed. Histological assessment revealed that the colonic mucosa of the control group remained intact, with preserved crypt architecture and an absence of inflammatory infiltration. In contrast, the DGP group showed pronounced mucosal lesions, including ulceration, congestion, extensive inflammatory cell infiltration, and significant goblet cell loss (Fig. **2A**). Both MFZLZD-H and Mos markedly attenuated these pathological changes (Fig. **2A**). The intestinal mucosal barrier, composed of closely connected epithelial cells and a protective mucus layer, is the first line of defense against pathogenic microorganisms [15]. We further evaluated colonic goblet cells using AB-PAS staining. While the control group displayed normal goblet cell morphology and a continuous mucus layer, the DGP group showed severe goblet cell depletion and mucus layer disintegration; these defects were substantially reversed following intervention with MFZLZD-H or Mos (Fig. **2B**). Furthermore, the expression of tight junction proteins ZO-1 and Occludin was notably decreased in the colonic tissues of the DGP group compared to controls. However, both MFZLZD-H and Mos effectively restored the levels of these proteins in the DGP group (Figs. **2C** and **D**). Taken together, these findings suggest that the therapeutic effects of MFZLZD on DGP may be mediated by the restoration of the colonic mucosal barrier.

### 3.3 MFZLZD Reshaped the Gut Microbiota Composition in DGP Rats

The human body is colonized by a vast microbial ecosystem, with over 70% of these microorganisms residing in the colon. These microbes act as a critical biological barrier against pathogens and perform essential metabolic functions [16]. Consequently, microbial dysbiosis is closely associated with various human diseases. To evaluate the impact of MFZLZD on the gut microbiota in DGP, 16S rRNA sequencing was performed on rat fecal samples. A total of 441 shared ASVs were identified across the three groups, while 836, 811, and 881 unique ASVs were found in the control, DGP, and DGP + MFZLZD-H groups, respectively (Fig. **3A**). The rank abundance curve exhibited a broad horizontal distribution, and the rarefaction curve reached a plateau, indicating high species richness and sufficient sampling depth (Fig. **3B**). In α-diversity analysis, the Good’s coverage was near 1.0, confirming adequate sequencing saturation (Fig. **3C**). Moreover, the Shannon and Simpson indices were significantly elevated in the DGP group compared to the Control and DGP + MFZLZD-H groups, although no significant differences were observed in ACE, Chao1, or observed species indices among the three groups (Fig. **3C**). The Simpson index in the DGP + MFZLZD-H group was notably lower than that of the control group, whereas the Shannon index showed no significant difference between these two groups (Fig. **3C**). These results suggested that DGP is associated with altered microbial diversity, which was partially restored following MFZLZD-H treatment. In β-diversity analysis, a clear separation was observed among the three groups (Fig. **3D**). MFZLZD substantially reshaped the overall microbiota structure in DGP rats.

Taxonomic profiling at the phylum and genus levels was further performed to elucidate specific microbial shifts. *Firmicutes* and *Bacteroidota* were the major gut microbiota at the phylum level (Fig. **4A**). At the genus level, the relative abundance of the *(Eubacterium) coprostanoligenes group*, *Akkermansia*, *Clostridia UCG-014*, *Muribaculaceae*, and *Ruminococcus* was decreased, while *Bacteroides*, *Blautia*, and *Romboutsia* increased in the DGP group compared to controls (Fig. **4B**). Compared to the DGP group, the relative abundance of the *(Eubacterium) coprostanoligenes group*, *Akkermansia*, *Muribaculaceae*, *Romboutsia*, and *Ruminococcus* was increased, while *Bacteroides* and *Blautia* decreased in the DGP + MFZLZD-H group (Fig. **4B**). LEfSe analysis (LDA score > 2 and *P* < 0.05) was conducted to identify differential gut microbiota composition between groups. There were 12 differential gut microbiota at the phylum level, 15 at the class level, 29 at the order level, 44 at the family level, 87 at the genus level, and 46 at the species level between the control and DGP groups (Fig. **5A**). Moreover, 11 differential gut microbiota at the phylum level, 19 at the class level, 38 at the order level, 50 at the family level, 98 at the genus level, and 36 at the species level were found between the DGP and DGP + MFZLZD-H groups (Fig. **5B**). After intersecting the differential gut microbiota, 33 key gut microbiota were identified, including *Absiella argi*, *Alistipes indistinctus*, *Alistipes obesi*, *Alistipes shahii*, *Anaerobiospirillum succiniciproducens*, *Anaerotruncus sp*, *Bacteroides clarus*, *Bacteroides intestinalis*, *Bacteroides vulgatus*, *Butyricimonas synergistica*, *Corynebacterium kutscheri*, *Corynebacterium stationis*, *Intestinimonas butyriciproducens*, *Lachnospiraceae bacterium*, *Lysinibacillus alkalisoli*, *Odoribacter splanchnicus*, *Parabacteroides merdae*, *Streptococcus merionis*, *bacterium*, *gut metagenome*, *human gut*, *uncultured Allobaculum*, *uncultured prokaryote*, *Helicobacter rodentium*, *Lactobacillus intestinalis*, *Ruminococcus flavefaciens*, *Streptococcus agalactiae*, *Clostridium leptum*, *uncultured Bacteroidales*, *uncultured Clostridia*, *uncultured Clostridiales*, *uncultured Clostridium*, and *uncultured bacterium* (Fig. **5C**).

## 4 DISCUSSION

DGP is a prevalent gastrointestinal complication of diabetic patients, characterized by non-specific clinical manifestations that render diagnosis challenging and leave its underlying pathogenesis elusive [3]. The present investigation demonstrates that MFZLZD effectively alleviates DGP symptoms, reduces colonic mucosal injury, and reshapes the gut microbiota in model rats. Specifically, 33 key gut microbiota were identified between groups.

DGP is fundamentally characterized by impaired gastrointestinal motility, presenting clinically as nausea, vomiting, early satiety, abdominal distension, and weight loss [17]. Li *et al*. have revealed that DGP rats exhibited reduced body weight, delayed gastric emptying, reduced intestinal propulsion, and structural abnormalities in gastric tissue [18]. Consistently, our data show that DGP rats underwent a progressive decline in body weight following STZ induction. Additionally, the gastric emptying rate, peristalsis frequency, and gastric blood flow were significantly decreased in DGP rats, accompanied by gastric mucosal damage. These observations collectively validate the successful establishment of the DGP model.

While conventional therapies are available, clinical evidence indicates that many DGP patients remain refractory to standard treatments. Conversely, TCM demonstrated significant clinical efficacy in managing DGP [19]. FZLZD is a well-established TCM formula, and its preparations have been used for decades to manage gastrointestinal diseases [20, 21]. Similarly, our experimental results showed that MFZLZD treatment was associated with improvements in body weight, gastric functional parameters, and histopathological damage in DGP rats.

The intestinal epithelial barrier comprises a monolayer of tightly packed epithelial cells and represents the most extensive mucosal interface in the human body. This epithelial layer, together with the mucus layer, isolates the intestinal lumen contents from the mucosal lamina propria, and its compromise is critically linked to the pathogenesis of various gastrointestinal diseases [22]. We found that DGP rats showed manifest colonic mucosal injury, accompanied by inflammatory infiltration and goblet cell loss, while MFZLZD alleviated these pathological changes. Similar to our results, research has shown that Fuzi Lizhong Pill might repair the colonic mucosal barrier by inhibiting inflammation, thereby exerting an anti-ulcerative colitis effect [20]. Under pathological conditions, reduced expression of tight junction proteins is frequently linked to inflammatory cytokines (*e.g.*, tumor necrosis factor-alpha), which activate NF-κB signaling, as well as to reactive oxygen species (ROS)-induced phosphorylation of these proteins [23]. We found that MFZLZD increased Occludin and ZO-1 levels in the colonic tissues of DGP rats. Consistent with this, Li *et al*. reported that Fuzi Lizhong pill inhibits the expression of TNF-α and IL-6 and promotes the expression of tight junction proteins in ulcerative colitis by downregulating the PI3K/Akt/NF-κB pathway [20]. Another study found that the Fuzi Lizhong pill exhibits anti-oxidative, anti-inflammatory, and immunomodulatory effects in Spleen-Yang deficiency [24]. Therefore, these changes in Occludin and ZO-1 expression may be associated with alterations in inflammatory responses and oxidative stress in DGP rats following MFZLZD treatment, though the definitive causal pathways remain to be established.

Various microorganisms colonize the skin and mucous membranes, with the vast majority residing within the gastrointestinal tract. The intricate crosstalk between the intestinal epithelial barrier, gut microbiota, and immune cells is pivotal for maintaining the host's immune defense [25]. Conversely, microbial dysbiosis may compromise the intestinal barrier and predispose the host to various pathologies [25]. We found that the diversity of the gut microbiota was significantly increased in DGP rats and was partially restored after MFZLZD treatment. Specifically, the relative abundance of the *(Eubacterium) coprostanoligenes group*, *Akkermansia*, *Clostridia UCG-014*, *Muribaculaceae*, and *Ruminococcus* was decreased, while *Bacteroides*, *Blautia*, and *Romboutsia* increased in the DGP group compared to controls, reflecting a profound shift in the microbial landscape associated with DGP. Furthermore, the microbiota composition of the DGP + MFZLZD-H group was similar to that of the control group, reflecting a restoration of the microbial landscape. Consistent with this, Xue *et al*. found that magnoloside A, the main phenylethanoid glycoside in HouPo, accelerates the intestinal emptying rate and regulates the composition of gut microbiota in rats with functional dyspepsia [26]. These findings suggest that the therapeutic effects of MFZLZD on intestinal mucosal injury and DGP symptoms are accompanied by a distinct shift in gut microbiota composition. Nevertheless, the mechanisms underlying these microbiota changes were not directly investigated in this study. Generally, herbal medicines inhibit the adhesion of pathogenic bacteria to intestinal epithelial cells and suppress their intracellular invasion and intestinal colonization. Concurrently, herbal polysaccharides, glycosides, and carbohydrates serve as prebiotics that selectively promote the proliferation of probiotics. Once enriched, these probiotics and their metabolites (such as SCFAs) activate diverse signaling pathways, exerting both localized gastrointestinal and systemic therapeutic effects [27]. However, the specific mechanisms by which MFZLZD influences the gut microbiome in DGP remain unclear and require further investigation.

This study has several limitations. Firstly, there was no further exploration of which active component in MFZLZD played a major role in DGP treatment. Additionally, the relatively small sample size may limit the statistical robustness of the study, and larger cohorts are needed to validate these findings. Moreover, the investigation of the gut microbiota is only based on 16S rRNA sequencing and lacks a comprehensive exploration of its functions and metabolic features. Furthermore, we have discussed the possible mechanisms by which MFZLZD regulates tight junction proteins and the gut microbiota in DGP, but these mechanisms were not experimentally verified.

## CONCLUSION

This study demonstrates that MFZLZD alleviates DGP symptoms by mitigating gastric dysfunction and restoring the intestinal mucosal barrier. Notably, the therapeutic effect of MFZLZD was accompanied by a significant remodeling of the gut microbiota composition, characterized by the normalization of microbial diversity and alterations in specific bacterial taxa. These findings provide a potential therapeutic framework for DGP management and lay the foundation for future research into the interplay between MFZLZD, the gut microbiota, and intestinal barrier function.

## Figures and Tables

**Fig. (1):**
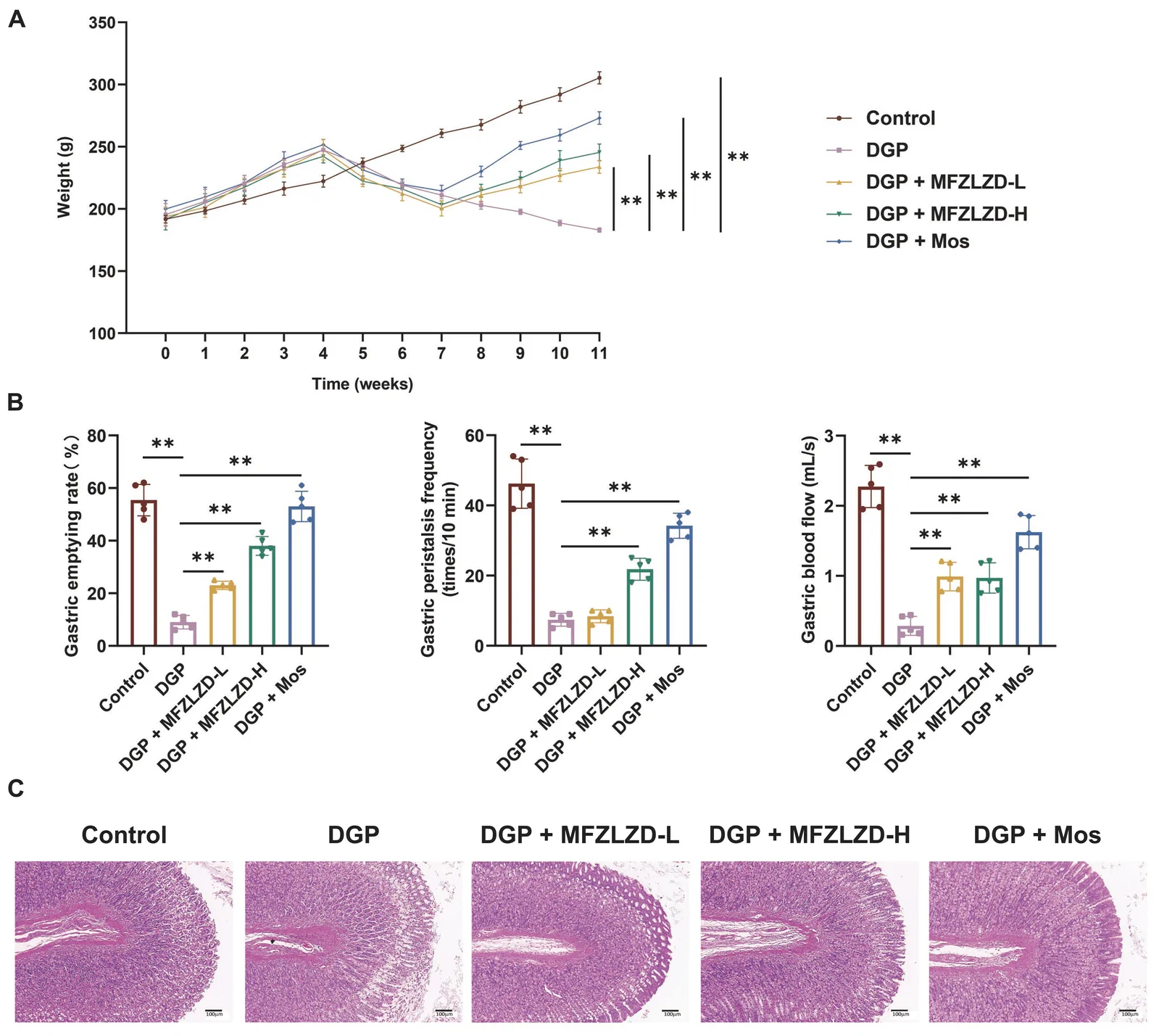
Modified Fuzi Lizhong decoction (MFZLZD) alleviated the symptoms of diabetic gastroparesis (DGP) rats. (**A**) The body weight of rats (n = 5). (**B**) The gastric emptying rate, gastric peristalsis frequency, and gastric blood flow of rats (n = 5). (**C**) Representative images of HE staining of gastric tissues (n = 3). Scale bar = 100 μm. **P < 0.01.

**Fig. (2):**
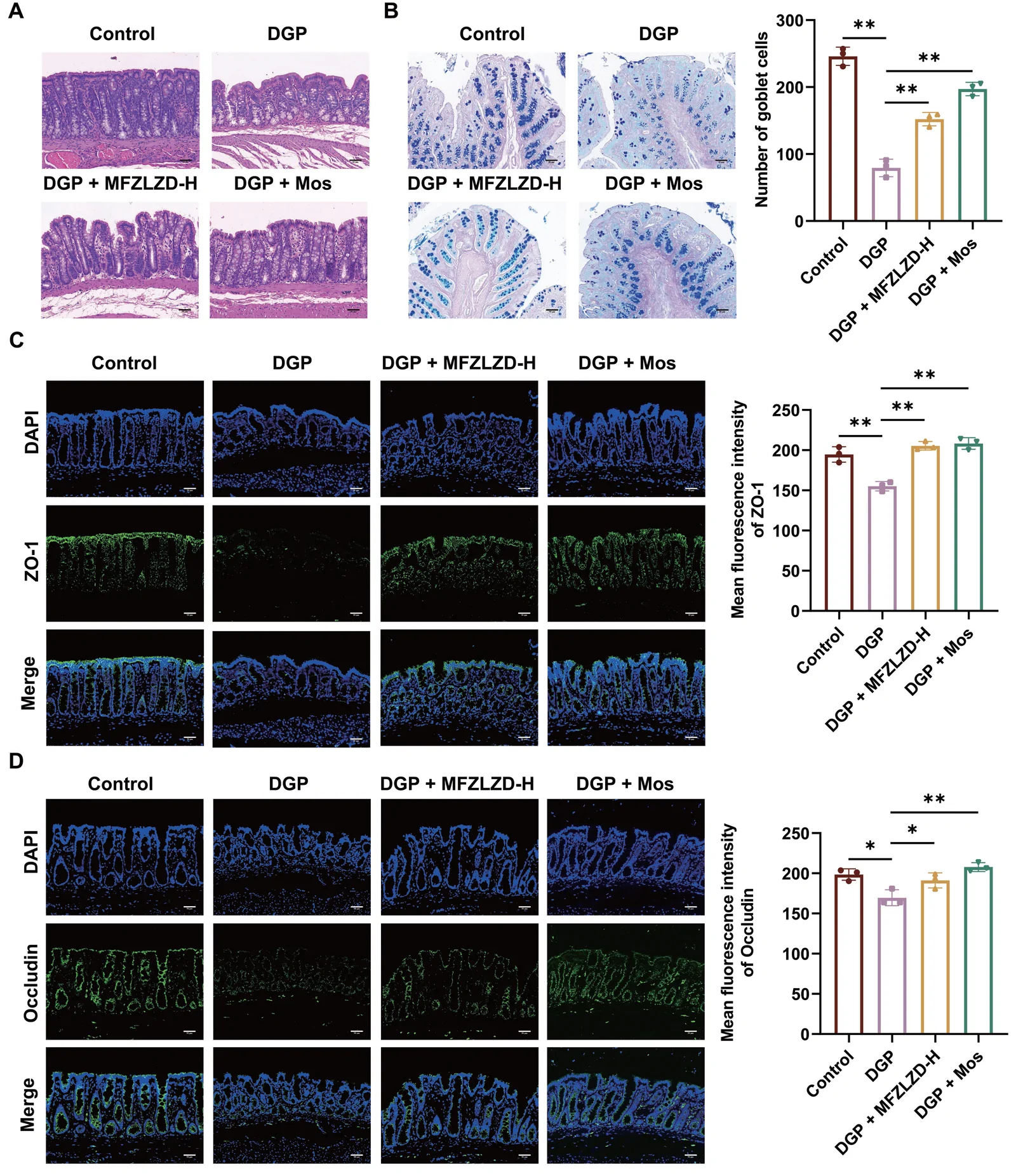
MFZLZD ameliorated intestinal mucosal barrier injury in DGP rats. (**A**) Representative images of HE staining of colonic tissues. Scale bar = 50 μm. (**B**) Representative images of AB-PAS staining of colonic tissues. Scale bar = 50 μm. (**C**) The expression of ZO-1 in colonic tissues was detected by immunofluorescence. Scale bar = 50 μm. (**D**) The expression of Occludin in colonic tissues was detected by immunofluorescence. Scale bar = 50 μm. n = 3. *P < 0.05, **P < 0.01.

**Fig. (3):**
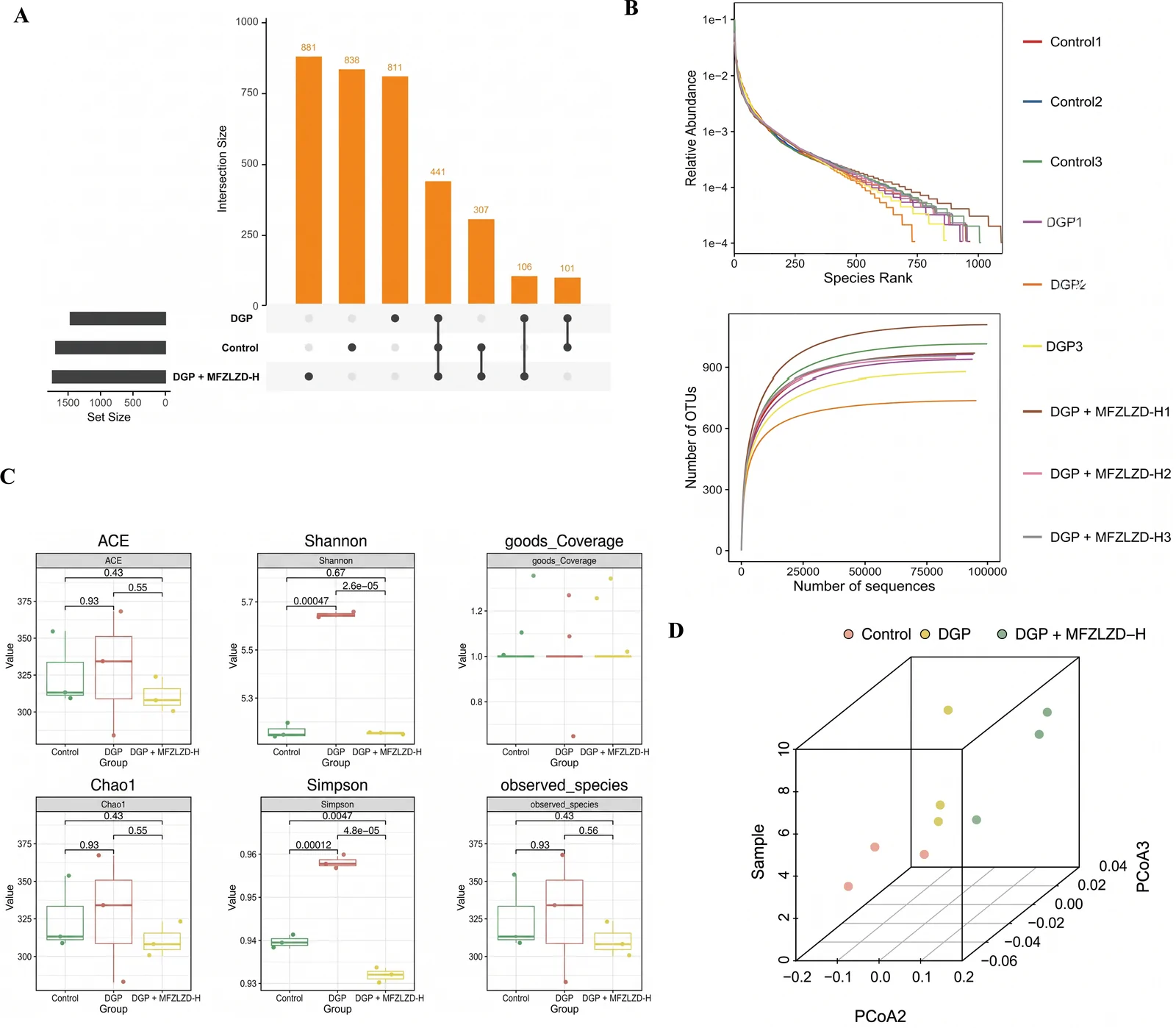
MFZLZD improved the gut microbiota dysbiosis caused by DGP. (**A**) Overlapping and unique ASVs among groups. (**B**) The rank abundance curve and rarefaction curve of the gut microbiota. (**C**) α diversity was evaluated using ACE, Chao1, Shannon, Simpson, observed species, and Good’s coverage. (**D**) β diversity of the gut microbiota was compared through PCoA analysis. n = 5.

**Fig. (4):**
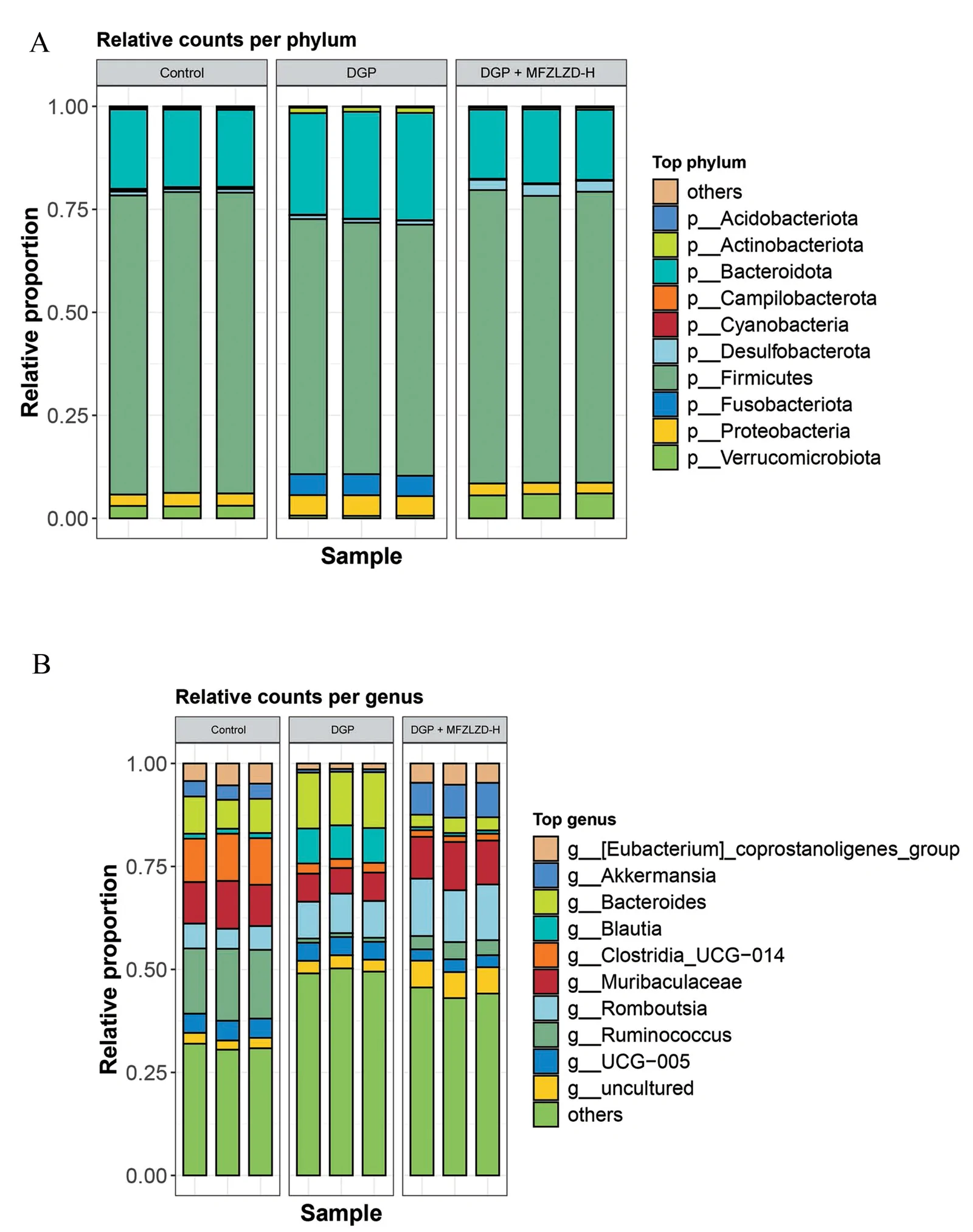
Composition of gut microbiota at the phylum and genus levels. Relative abundance of gut microbiota at the phylum (**A**) and genus (**B**) levels in each group. n = 5.

**Fig. (5):**
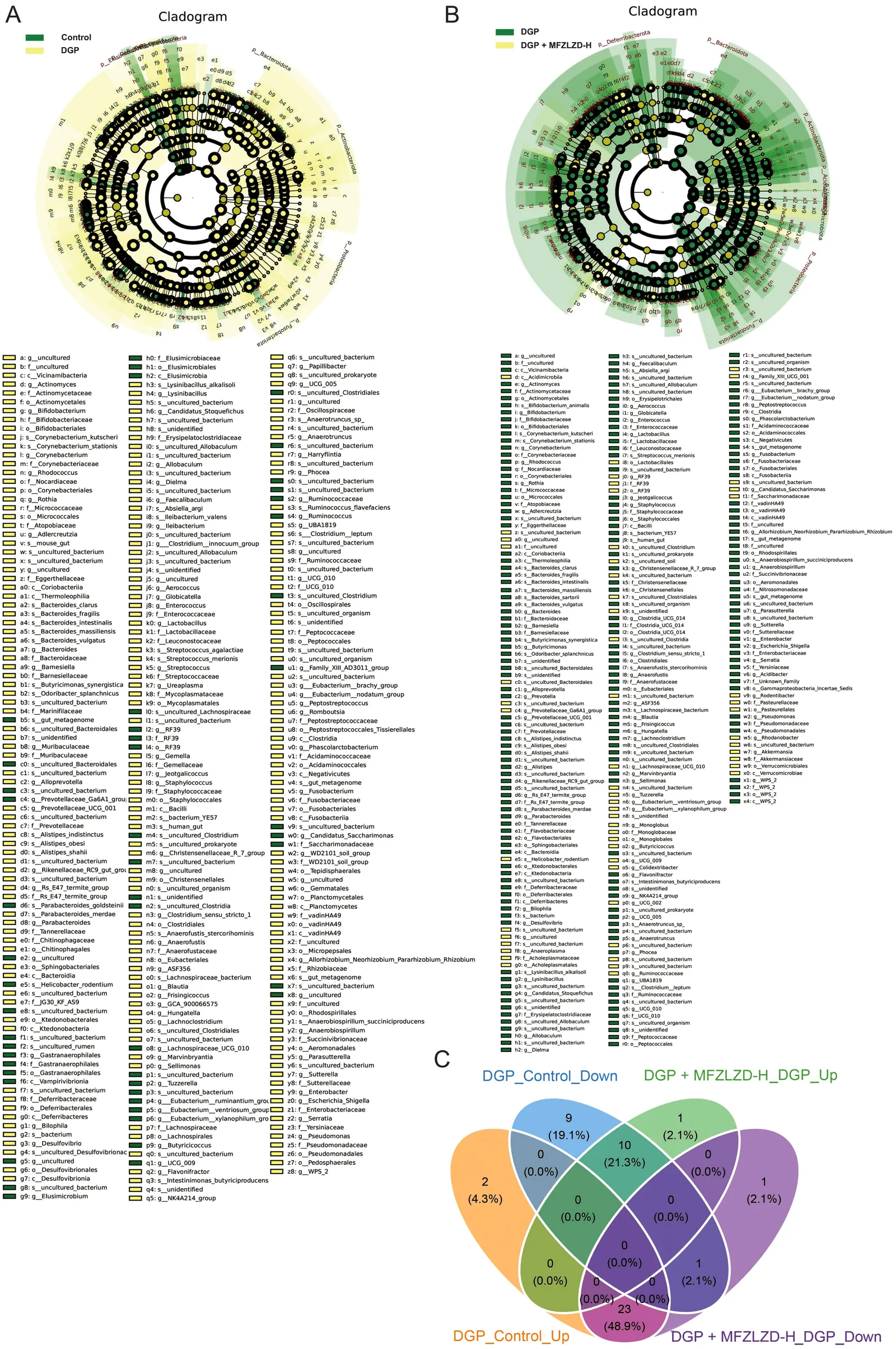
Identification of key differential gut microbiota. LEfSe analysis identified differential gut microbiota between the control and DGP groups (**A**), and between the DGP and DGP + MFZLZD-H groups (**B**). (**C**) The key differential gut microbiota were identified through intersection analysis. n = 5.

## Data Availability

The data that support the findings of this study are available from the corresponding author, [Zhengang Wang], on special request.

## LIST OF ABBREVIATIONS

DGP: Diabetic Gastroparesis
MFZLZD: Modified Fuzi Lizhong Decoction
SCFAs: Short-Chain Fatty Acids
TCM: Traditional Chinese Medicine
STZ: Streptozotocin
Mos: Mosapride
ASV: Amplicon Sequence Variants

## References

[1] ElSayed N.A., Aleppo G., Bannuru R.R., Bruemmer D., Collins B.S., Ekhlaspour L., Gaglia J.L., Hilliard M.E., Johnson E.L., Khunti K., Lingvay I., Matfin G., McCoy R.G., Perry M.L., Pilla S.J., Polsky S., Prahalad P., Pratley R.E., Segal A.R., Seley J.J., Selvin E., Stanton R.C., Gabbay R.A. (2024). 2. Diagnosis and classification of diabetes: Standards of care in diabetes—2024.. Diabetes Care.

[2] Xu S., Fu H., Weng S., Gu X., Li J. (2023). Derivation and comprehensive analysis of ageing-related genes in intervertebral disc degeneration for prediction and immunology.. Mech. Ageing Dev..

[3] Zhou J., Ran S.L., Zhao Y.C. (2024). Diabetic gastroparesis: A disease for which long-term therapeutic benefits are difficult to obtain.. Endokrynol. Pol..

[4] Ahmed M.S.O., Forde H., Smith D. (2023). Diabetic gastroparesis: Clinical features, diagnosis and management.. Ir. J. Med. Sci..

[5] Adak A., Khan M.R. (2019). An insight into gut microbiota and its functionalities.. Cell. Mol. Life Sci..

[6] Wauters L., Li H., Talley N.J. (2022). Editorial: Disruption of the microbiota-gut-brain axis in functional dyspepsia and gastroparesis: Mechanisms and clinical implications.. Front. Neurosci..

[7] Lau W.L., Tran T., Rhee C.M., Kalantar-Zadeh K., Vaziri N.D. (2021). Diabetes and the gut microbiome.. Semin. Nephrol..

[8] Mandarino F.V., Sinagra E., Barchi A., Verga M.C., Brinch D., Raimondo D., Danese S. (2023). Gastroparesis: The complex interplay with microbiota and the role of exogenous infections in the pathogenesis of the disease.. Microorganisms.

[9] Zheng X., Zhang Y., Tan Y., Li Y., Xue Q., Li H., Zhang X., Pan Y., Xu J., Zhang J. (2024). *Alpinia officinarum* Hance extract ameliorates diabetic gastroparesis by regulating SCF/c-kit signaling pathway and rebalancing gut microbiota.. Fitoterapia.

[10] Zhang Y.X., Zhang Y.J., Miao R.Y., Fang X.Y., Wei J.H., Wei Y., Lin J.R., Tian J.X. (2023). Effectiveness and safety of traditional Chinese medicine decoction for diabetic gastroparesis: A network meta-analysis.. World J. Diabetes.

[11] Bai L., Liu Y., Ji Y. (2022). Effect of modified fuzi lizhong decoction on diabetic gastroparesis.. Tangniaobing Xinshijie.

[12] Zhen Z., Xia L., You H., Jingwei Z., Shasha Y., Xinyi W., Wenjing L., Xin Z., Chaomei F. (2021). An integrated gut microbiota and network pharmacology study on fuzi-lizhong pill for treating diarrhea-predominant irritable bowel syndrome.. Front. Pharmacol..

[13] Zhou Y., Wu J., Wang H., Feng W., Peng F., Zhang R., Yan H., Liu J., Tan Y., Peng C. (2024). Fuzi lizhong pills alter microbial community compositions and metabolite profiles in ulcerative colitis rat with spleen-kidney yang deficiency syndrome.. J. Ethnopharmacol..

[14] Crimmins S., Smiley R., Preston K., Yau A., Mccallum R., Ali M.S. (2016). Increased expression of pyloric ERβ is associated with diabetic gastroparesis in streptozotocin-induced male diabetic rats.. Gastroenterol. Res..

[15] Cai R., Cheng C., Chen J., Xu X., Ding C., Gu B. (2020). Interactions of commensal and pathogenic microorganisms with the mucus layer in the colon.. Gut Microbes.

[16] Schippa S., Conte M. (2014). Dysbiotic events in gut microbiota: Impact on human health.. Nutrients.

[17] Zhang Y.X., Zhang Y.J., Li M., Tian J.X., Tong X.L. (2024). Common pathophysiological mechanisms and treatment of diabetic gastroparesis.. J. Neurogastroenterol. Motil..

[18] Li H., Wang Y., Tian Y., Tian F., Xing Z., Wang Y., Yan M., Gong Y. (2022). *Atractylodes chinensis* volatile oil up-regulated IGF-1 to improve diabetic gastroparesis in rats.. Iran. J. Basic Med. Sci..

[19] Pang B., Zhou Q., Li J.L., Zhao L.H., Tong X.L. (2014). Treatment of refractory diabetic gastroparesis: Western medicine and traditional Chinese medicine therapies.. World J. Gastroenterol..

[20] Li Y., Tian Y., Zhu L., Lin H., Zhao X., Liu C., Lv Y., Wang Z., Zuo Z., Wang J., Wang Z. (2025). Fuzi Lizhong Pill inhibited inflammatory response and promoted colon mucosal healing in dextran sulfate sodium-induced ulcerative colitis mice by down-regulating PI3K/AKT/NF-κB signaling pathway.. J. Ethnopharmacol..

[21] Li Y., Zuo Z., Tian Y., Zhao X., Liu C., Yu S., Pei H., Tian S., Wang Z. (2024). Research progress in the treatment of ulcerative colitis by Fuzi Lizhong Prescription.. Chin. J. Integr. Tradit. West. Med. Dig..

[22] Ahluwalia B., Magnusson M.K., Öhman L. (2017). Mucosal immune system of the gastrointestinal tract: Maintaining balance between the good and the bad.. Scand. J. Gastroenterol..

[23] Chelakkot C., Ghim J., Ryu S.H. (2018). Mechanisms regulating intestinal barrier integrity and its pathological implications.. Exp. Mol. Med..

[24] Zhang Z., Yang S., Lin X., Huang Y., Wei X., Zhou J., Li R., Deng B., Fu C. (2021). Metabolomics of Spleen-Yang deficiency syndrome and the therapeutic effect of Fuzi Lizhong pill on regulating endogenous metabolism.. J. Ethnopharmacol..

[25] Takiishi T., Fenero C.I.M., Câmara N.O.S. (2017). Intestinal barrier and gut microbiota: Shaping our immune responses throughout life.. Tissue Barriers.

[26] Xue Z., Wu C., Wei J., Xian M., Wang T., Yang B., Chen M. (2019). An orally administered magnoloside A ameliorates functional dyspepsia by modulating brain-gut peptides and gut microbiota.. Life Sci..

[27] Xu J., Chen H.B., Li S.L. (2017). Understanding the molecular mechanisms of the interplay between herbal medicines and gut microbiota.. Med. Res. Rev..

